# Lasers, stem cells, and COPD

**DOI:** 10.1186/1479-5876-8-16

**Published:** 2010-02-16

**Authors:** Feng Lin, Steven F Josephs, Doru T Alexandrescu, Famela Ramos, Vladimir Bogin, Vincent Gammill, Constantin A Dasanu, Rosalia De Necochea-Campion, Amit N Patel, Ewa Carrier, David R Koos

**Affiliations:** 1 Entest BioMedical, San Diego, CA, USA; 2 Georgetown Dermatology, Washington DC, USA; 3 Cromos Pharma Services, Longview, WA, USA; 4 Center for the Study of Natural Oncology, Del Mar, CA, USA; 5 Department of Hematology and Medical Oncology, St Francis Hospital and Medical Center, Hartford, CT, USA; 6 Moores Cancer Center, University of California San Diego, CA, USA; 7 Department of Cardiothoracic Surgery, University of Utah, Salt Lake City, UT, USA

## Abstract

The medical use of low level laser (LLL) irradiation has been occurring for decades, primarily in the area of tissue healing and inflammatory conditions. Despite little mechanistic knowledge, the concept of a non-invasive, non-thermal intervention that has the potential to modulate regenerative processes is worthy of attention when searching for novel methods of augmenting stem cell-based therapies. Here we discuss the use of LLL irradiation as a "photoceutical" for enhancing production of stem cell growth/chemoattractant factors, stimulation of angiogenesis, and directly augmenting proliferation of stem cells. The combination of LLL together with allogeneic and autologous stem cells, as well as post-mobilization directing of stem cells will be discussed.

## Introduction (Personal Perspective)

We came upon the field of low level laser (LLL) therapy by accident. One of our advisors read a press release about a company using this novel technology of specific light wavelengths to treat stroke. Given the possible role of stem cells in post-stroke regeneration, we decided to cautiously investigate. As a background, it should be said that our scientific team has been focusing on the area of cord blood banking and manufacturing of disposables for processing of adipose stem cells for the past 3 years. Our board has been interested in strategically refocusing the company from services-oriented into a more research-focused model. An unbiased exploration into the various degenerative conditions that may be addressed by our existing know-how led us to explore the condition of chronic obstructive pulmonary disease (COPD), an umbrella term covering chronic bronchitis and emphysema, which is the 4^th ^largest cause of death in the United States. As a means of increasing our probability of success in treatment of this condition, the decision was made to develop an adjuvant therapy that would augment stem cell activity. The field of LLL therapy attracted us because it appeared to be relatively unexplored scientific territory for which large amounts of clinical experience exist. Unfortunately, it was difficult to obtain the cohesive "state-of-the-art" description of the molecular/cellular mechanisms of this therapy in reviews that we have searched. Therefore we sought in this mini-review to discuss what we believe to be relevant to investigators attracted by the concept of "regenerative photoceuticals". Before presenting our synthesis of the field, we will begin by describing our rationale for approaching COPD with the autologous stem cell based approaches we are developing.

## COPD as an Indication for Stem Cell Therapy

COPD possesses several features making it ideal for stem cell based interventions: a) the quality of life and lack of progress demands the ethical exploration of novel approaches. For example, bone marrow stem cells have been used in over a thousand cardiac patients with some indication of efficacy [1,2]. Adipose-based stem cell therapies have been successfully used in thousands of race-horses and companion animals without adverse effects [3], as well as numerous clinical trials are ongoing and published human data reports no adverse effects (reviewed in ref [4]). Unfortunately, evaluation of stem cell therapy in COPD has lagged behind other areas of regenerative investigation; b) the underlying cause of COPD appears to be inflammatory and/or immunologically mediated. The destruction of alveolar tissue is associated with T cell reactivity [5,6], pathological pulmonary macrophage activation [7], and auto-antibody production [8]. Mesenchymal stem cells have been demonstrated to potently suppress autoreactive T cells [9,10], inhibit macrophage activation [11], and autoantibody responses [12]. Additionally, mesenchymal stem cells can be purified in high concentrations from adipose stromal vascular tissue together with high concentrations of T regulatory cells [4], which in animal models are approximately 100 more potent than peripheral T cells at secreting cytokines therapeutic for COPD such as IL-10 [13,14]. Additionally, use of adipose derived cells has yielded promising clinical results in autoimmune conditions such as multiple sclerosis [4]; and c) Pulmonary stem cells capable of regenerating damaged parenchymal tissue have been reported [15]. Administration of mesenchymal stem cells into neonatal oxygen-damaged lungs, which results in COPD-like alveoli dysplasia, has been demonstrated to yield improvements in two recent publications [16,17].

Based on the above rationale for stem cell-based COPD treatments, we began our exploration into this area by performing several preliminary experiments and filing patents covering combination uses of stem cells with various pharmacologically available antiinflammatories, as well as methods of immune modulation. These have served as the basis for two of our pipeline candidates, ENT-111, and ENT-894. As a commercially-oriented organization, we needed to develop a therapeutic candidate that not only has a great potential for efficacy, but also can be easily implemented as part of the standard of care. Our search led us to the area of low level laser (LLL) therapy. From our initial perception as neophytes to this field, the area of LLL therapy has been somewhat of a medical mystery. A pubmed search for "low level laser therapy" yields more than 1700 results, yet before stumbling across this concept, none of us, or our advisors, have ever heard of this area of medicine.

On face value, this field appeared to be somewhat of a panacea: clinical trials claiming efficacy for conditions ranging from alcoholism [18], to sinusitis [19], to ischemic heart disease [20]. Further confusing was that many of the studies used different types of LLL-generating devices, with different parameters, in different model systems, making comparison of data almost impossible. Despite this initial impression, the possibility that a simple, non-invasive methodology could exist that augments regenerative potential in a tissue-focused manner became very enticing to us. Specific uses envisioned, for which intellectual property was filed included using light to concentrate stem cells to an area of need, to modulate effects of stem cells once they are in that specific area, or even to use light together with other agents to modulate endogenous stem cells.

The purpose of the current manuscript is to overview some of the previous work performed in this area that was of great interest to our ongoing work in regenerative medicine. We believe that greater integration of the area of LLL with current advancements in molecular and cellular biology will accelerate medical progress. Unfortunately, in our impression to date, this has been a very slow process.

## What is Low Level Laser Irradiation?

Lasers (Light amplification by stimulated emission of radiation) are devices that typically generate electromagnetic radiation which is relatively uniform in wavelength, phase, and polarization, originally described by Theodore Maiman in 1960 in the form of a ruby laser [21]. These properties have allowed for numerous medical applications including uses in surgery, activation of photodynamic agents, and various ablative therapies in cosmetics that are based on heat/tissue destruction generated by the laser beam [22-24]. These applications of lasers are considered "high energy" because of their intensity, which ranges from about 10-100 Watts. The subject of the current paper will be another type of laser approach called low level lasers (LLL) that elicits effects through non-thermal means. This area of investigation started with the work of Mester et al who in 1967 reported non-thermal effects of lasers on mouse hair growth [25]. In a subsequent study [26], the same group reported acceleration of wound healing and improvement in regenerative ability of muscle fibers post wounding using a 1 J/cm^2 ^ruby laser. Since those early days, numerous in vitro and in vivo studies have been reported demonstrating a wide variety of therapeutic effects involving LLL, a selected sample of which will be discussed below. In order to narrow our focus of discussion, it is important to first begin by establishing the current definition of LLL therapy. According to Posten et al [27], there are several parameters of importance: a) Power output of laser being 10^-3 ^to 10^-1 ^Watts; b) Wavelength in the range of 300-10,600 nm; c) Pulse rate from 0, meaning continuous to 5000 Hertz (cycles per second); d) intensity of 10^-2^-10 W/cm(2) and dose of 0.01 to 100 J/cm^2^. Most common methods of administering LLL radiation include lasers such as ruby (694 nm), Ar (488 and 514 nm), He-Ne (632.8 nm), Krypton (521, 530, 568, and 647 nm), Ga-Al-As (805 or 650 nm), and Ga-As (904 nm). Perhaps one of the most distinguishing features of LLL therapy as compared to other photoceutical modalities is that effects are mediated not through induction of thermal effects but rather through a process that is still not clearly defined called "photobiostimulation". It appears that this effect of LLL is not depend on coherence, and therefore allows for use of non-laser light generating devices such as inexpensive Light Emitting Diode (LED) technology [28].

To date several mechanisms of biological action have been proposed, although none are clearly established. These include augmentation of cellular ATP levels [29], manipulation of inducible nitric oxide synthase (iNOS) activity [30,31], suppression of inflammatory cytokines such as TNF-alpha, IL-1beta, IL-6 and IL-8 [32-36], upregulation of growth factor production such as PDGF, IGF-1, NGF and FGF-2 [36-39], alteration of mitochondrial membrane potential [29,40-42] due to chromophores found in the mitochondrial respiratory chain [43,44] as reviewed in [45], stimulation of protein kinase C (PKC) activation [46], manipulation of NF-κB activation [47], direct bacteriotoxic effect mediated by induction of reactive oxygen species (ROS) [48], modification of extracellular matrix components [49], inhibition of apoptosis [29], stimulation of mast cell degranulation [50], and upregulation of heat shock proteins [51]. Unfortunately these effects have been demonstrated using a variety of LLL devices in non-comparable models. To add to confusion, dose-dependency seems to be confined to such a narrow range or does not seem to exist in that numerous systems therapeutic effects disappear with increased dose.

## In vitro studies of LLL

In areas of potential phenomenology, it is important to begin by assessing in vitro studies reported in the literature in which reproducibility can be attained with some degree of confidence, and mechanistic dissection is simpler as compared with in vivo systems. In 1983, one of the first studies to demonstrate in vitro effects of LLL was published. The investigators used a helium neon (He-Ne) laser to generate a visible red light at 632.8 nm for treatment of porcine granulosa cells. The paper described upregulation of metabolic and hormone-producing activity of the cells when exposed for 60 seconds to pulsating low power (2.8 mW) irradiation [52]. The possibility of modulating biologically-relevant signaling proteins by LLL was further assessed in a study using an energy dose of 1.5 J/cm^2 ^in cultured keratinocytes. Administration of He-Ne laser emitted light resulted in upregulated gene expression of IL-1 and IL-8 [53]. Production of various growth factors in vitro suggests the possibility of enhanced cellular mitogenesis and mobility as a result of LLL treatment. Using a diode-based method to generate a similar wavelength to the He-Ne laser (363 nm), Mvula et al reported in two papers that irradiation at 5 J/cm^2 ^of adipose derived mesenchymal stem cells resulted in enhanced proliferation, viability and expression of the adhesion molecule beta-1 integrin as compared to control [54,55]. In agreement with possible regenerative activity based on activation of stem cells, other studies have used an in vitro injury model to examine possible therapeutic effects. Migration of fibroblasts was demonstrated to be enhanced in a "wound assay" in which cell monolayers are scraped with a pipette tip and amount of time needed to restore the monolayer is used as an indicator of "healing". The cells exposed to 5 J/cm^2 ^generated by an He-Ne laser migrated rapidly across the wound margin indicating a stimulatory or positive influence of phototherapy. Higher doses (10 and 16 J/cm^2^) caused a decrease in cell viability and proliferation with a significant amount of damage to the cell membrane and DNA [56]. In order to examine whether LLL may positively affect healing under non-optimal conditions that mimic clinical situations treatment of fibroblasts from diabetic animals was performed. It was demonstrated that with the He-Ne laser dosage of 5 J/cm^2 ^fibroblasts exhibited an enhanced migration activity, however at 16 J/cm^2 ^activity was negated and cellular damage observed [57]. Thus from these studies it appears that energy doses from 1.5 J/cm^2 ^to 5 J/cm^2 ^are capable of eliciting "biostimulatory effects" in vitro in the He-Ne-based laser for adherent cells that may be useful in regeneration such as fibroblasts and mesenchymal stem cells.

Studies have also been performed in vitro on immunological cells. High intensity He-Ne irradiation at 28 and 112 J/cm^2 ^of human peripheral blood mononuclear cells, a heterogeneous population of T cells, B cells, NK cells, and monocytes has been described to induce chromatin relaxation and to augment proliferative response to the T cell mitogen phytohemaglutin [58]. In human peripheral blood mononuclear cells (PBMC), another group reported in two papers that interleukin-1 alpha (IL-1 alpha), tumor necrosis factor-alpha (TNF-alpha), interleukin-2 (IL-2), and interferon-gamma (IFN-gamma) at a protein and gene level in PBMC was increased after He-Ne irradiation at 18.9 J/cm^2 ^and decreased with 37.8 J/cm^2 ^[59,60]. Stimulation of human PBMC proliferation and murine splenic lymphocytes was also reported with He-Ne LLL [61,62]. In terms of innate immune cells, enhanced phagocytic activity of murine macrophages have been reported with energy densities ranging from 100 to 600 J/cm^2^, with an optimal dose of 200 J/cm^2 ^[63]. Furthermore, LLL has been demonstrated to augment human monocyte killing mycobacterial cells at similar densities, providing a functional correlation [64].

Thus from the selected in vitro studies discussed, it appears that modulation of proliferation and soluble factor production by LLL can be reliably reproduced. However the data may be to some extent contradictory. For example, the over-arching clinical rationale for use of LLL in conditions such as sinusitis [65], arthritis [66,67], or wound healing [68] is that treatment is associated with anti-inflammatory effects. However the in vitro studies described above suggested LLL stimulates proinflammatory agents such as TNF-alpha or IL-1 [59,60]. This suggests the in vivo effects of LLL may be very complex, which to some extent should not be surprising. Factors affecting LLL in vivo actions would include degree of energy penetration through the tissue, the various absorption ability of cells in the various tissues, and complex chemical changes that maybe occurring in paracrine/autocrine manner. Perhaps an analogy to the possible discrepancy between LLL effects in vitro versus in vivo may be made with the medical practice of extracorporeal ozonation of blood. This practice is similar to LLL therapy given that it is used in treatment of conditions such as atherosclerosis, non-healing ulcers, and various degenerative conditions, despite no clear mechanistic understanding [69-71]. In vitro studies have demonstrated that ozone is a potent oxidant and inducer of cell apoptosis and inflammatory signaling [72-74]. In contrast, in vivo systemic changes subsequent to administration of ozone or ozonized blood in animal models and patients are quite the opposite. Numerous investigators have published enhanced anti-oxidant enzyme activity such as elevations in Mg-SOD and glutathione-peroxidase levels, as well as diminishment of inflammation-associated pathology [75-78]. Regardless of the complexity of in vivo situations, the fact that reproducible, in vitro experiments, demonstrate a biological effect provided support for us that there is some basis for LLL and it is not strictly an area of phenomenology.

## Animal Studies with LLL

As early as 1983, Surinchak et al reported in a rat skin incision healing model that wounds exposed He-Ne radiation of fluency 2.2 J/cm^2 ^for 3 min twice daily for 14 days demonstrated a 55% increase in breaking strength over control rats. Interestingly, higher doses yielded poorer healing [79]. This application of laser light was performed directly on shaved skin. In a contradictory experiment, it was reported that rats irradiated for 12 days with four levels of laser light (0.0, 0.47, 0.93, and 1.73 J/cm^2^) a possible strengthening of wounds tension was observed at the highest levels of irradiation (1.73 J/cm^2^), however it did not reach significance when analyzed by resampling statistics [80]. In another wound-healing study Ghamsari et al reported accelerated healing in the cranial surface of teats in dairy cows by administration of He-Ne irradiation at 3.64 J/cm^2 ^dose of low-level laser, using a helium-neon system with an output of 8.5 mW, continuous wave [81]. Collagen fibers in LLL groups were denser, thicker, better arranged and more continuous with existing collagen fibers than those in non-LLL groups. The mean tensile strength was significantly greater in LLL groups than in non-LLL groups [82]. In the random skin flap model, the use of He-Ne laser irradiation with 3 J/cm^2 ^energy density immediately after the surgery and for the four subsequent days was evaluated in 4 experimental groups: Group 1 (control) sham irradiation with He-Ne laser; Group 2 irradiation by punctual contact technique on the skin flap surface; Group 3 laser irradiation surrounding the skin flap; and Group 4 laser irradiation both on the skin flap surface and around it. The percentage of necrotic area of the four groups was determined on day 7-post injury. The control group had an average necrotic area of 48.86%; the group irradiated on the skin flap surface alone had 38.67%; the group irradiated around the skin flap had 35.34%; and the group irradiated one the skin flap surface and around it had 22.61%. All experimental groups reached statistically significant values when compared to control [83]. Quite striking results were obtained in an alloxan-induced diabetes wound healing model in which a circular 4 cm^2 ^excisional wound was created on the dorsum of the diabetic rats. Treatment with He-Ne irradiation at 4.8 J/cm^2 ^was performed 5 days a week until the wound healed completely and compared to sham irradiated animals. The laser-treated group healed on average by the 18th day whereas, the control group healed on average by the 59th day [84].

In addition to mechanically-induced wounds, beneficial effects of LLL have been obtained in burn-wounds in which deep second-degree burn wounds were induced in rats and the effects of daily He-Ne irradiation at 1.2 and 2.4 J/cm^2 ^were assessed in comparison to 0.2% nitrofurazone cream. The number of macrophages at day 16, and the depth of new epidermis at day 30, was significantly less in the laser treated groups in comparison with control and nitrofurazone treated groups. Additionally, infections with S. epidermidis and S. aureus were significantly reduced [85].

While numerous studies have examined dermatological applications of LLL, which may conceptually be easier to perform due to ability to topically apply light, extensive investigation has also been made in the area of orthopedic applications. Healing acceleration has been observed in regeneration of the rat mid-cortical diaphysis of the tibiae, which is a model of post-injury bone healing. A small hole was surgically made with a dentistry burr in the tibia and the injured area and LLL was administered over a 7 or 14 day course transcutaneously starting 24 h from surgery. Incident energy density dosages of 31.5 and 94.5 J/cm^2 ^were applied during the period of the tibia wound healing. Increased angiogenesis was observed after 7 days irradiation at an energy density of 94.5 J/cm^2^, but significantly decreased the number of vessels in the 14-day irradiated tibiae, independent of the dosage [86]. In an osteoarthritis model treatment with He-Ne resulted in augmentation of heat shock proteins and pathohistological improvement of arthritic cartilage [87]. The possibility that a type of preconditioning response is occurring, which would involve induction of genes such as hemoxygenase-1 [88], remains to be investigated. Effects of LLL therapy on articular cartilage were confirmed by another group. The experiment consisted of 42 young Wistar rats whose hind limbs were operated on in order to immobilize the knee joint. One week after operation they were assigned to three groups; irradiance 3.9 W/cm2, 5.8 W/cm^2^, and sham treatment. After 6 times of treatment for another 2 weeks significantpreservation of articular cartilage stiffness with 3.9 and 5.8 W/cm^2 ^therapy was observed [89].

Muscle regeneration by LLL was demonstrated in a rat model of disuse atrophy in which eight-week-old rats were subjected to hindlimb suspension for 2 weeks, after which they were released and recovered. During the recovery period, rats underwent daily LLL irradiation (Ga-Al-As laser; 830 nm; 60 mW; total, 180 s) to the right gastrocnemius muscle through the skin. After 2-weeks the number of capillaries and fibroblast growth factor levels exhibited significant elevation relative to those of the LLL-untreated muscles. LLL treatment induced proliferation in satellite cells as detected by BRdU [90].

Other animal studies of LLL have demonstrated effects in areas that appear unrelated such as suppression of snake venom induced muscle death [91], decreasing histamine-induced vasospasms [92], inhibition of post-injury restenosis [93], and immune stimulation by thymic irradiation [94].

## Clinical Studies Using LLL

Growth factor secretion by LLL and its apparent regenerative activities have stimulated studies in radiation-induced mucositis. A 30 patient randomized trial of carcinoma patients treated by radiotherapy alone (65 Gy at a rate of 2 Gy/fraction, 5 fractions per week) without prior surgery or concomitant chemotherapy suffering from radiation-induced mucositis was performed using a He-Ne 60 mW laser. Grade 3 mucositis occured with a frequency of 35.2% in controls and at 7.6% of treated patients. Furthermore, a decrease in "severe pain" (grade 3) was observed in that 23.8% in the control group experienced this level of pain, as compared to 1.9% in the treatment group [95]. A subsequent study reported similar effects [96].

Healing ability of lasers was also observed in a study of patients with gingival flap incisions. Fifty-eight extraction patients had one of two gingival flap incisions lased with a 1.4 mW He-Ne (670 nm) at 0.34 J/cm^2^. Healing rates were evaluated clinically and photographically. Sixty-nine percent of the irradiated incisions healed faster than the control incisions. No significant difference in healing was noted when patients were compared by age, gender, race, and anatomic location of the incision [97]. Another study evaluating healing effects of LLL in dental practice examined 48 patients subjected to surgical removal of their lower third molars. Treated patients were administered Ga-Al-As diode generated 808 nm at a dose of 12 J. The study demonstrated that extraoral LLL is more effective than intraoral LLL, which was more effective than control for the reduction of postoperative trismus and swelling after extraction of the lower third molar [98].

Given the predominance of data supporting fibroblast proliferative ability and animal wound healing effects of LLL therapy, a clinical trial was performed on healing of ulcers. In a double-blinded fashion 23 diabetic leg ulcers from 14 patients were divided into two groups. Phototherapy was applied (<1.0 J/cm^2^) twice per week, using a Dynatron Solaris 705(R) LED device that concurrently emits 660 and 890 nm energies. At days 15, 30, 45, 60, 75, and 90 mean ulcer granulation and healing rates were significantly higher for the treatment group as compared to control. By day 90, 58.3% of the ulcers in the LLL treated group were fully healed and 75% achieved 90-100% healing. In the placebo group only one ulcer healed fully [68].

As previously mentioned, LLL appears to have some angiogenic activity. One of the major problems in coronary artery disease is lack of collateralization. In a 39 patient study advanced CAD, two sessions of irradiation of low-energy laser light on skin in the chest area from helium-neon B1 lasers. The time of irradiation was 15 minutes while operations were performed 6 days a week for one month. Reduction in Canadian Cardiology Society (CCS) score, increased exercise capacity and time, less frequent angina symptoms during the treadmill test, longer distance of 6-minute walk test and a trend towards less frequent 1 mm ST depression lasting 1 min during Holter recordings was noted after therapy [99].

Perhaps one of the largest clinical trials with LLL was the NEST trial performed by Photothera. In this double blind trial 660 stroke patients were recruited and randomized: 331 received LLL and 327 received sham. No prespecified test achieved significance, but a post hoc analysis of patients with a baseline National Institutes of Health Stroke Scale score of <16 showed a favorable outcome at 90 days on the primary end point (P < 0.044) [100]. Currently Photothera is in the process of repeating this trial with modified parameters.

## Relevance of LLL to COPD

A therapeutic intervention in COPD would require addressing the issues of inflammation and regeneration. Although approaches such as administration of bone marrow stem cells, or fat derived cellular components have both regenerative and anti-inflammatory activity in animal models, the need to enhance their potency for clinical applications can be seen in the recent Osiris's COPD trial interim data which reported no significant improvement in pulmonary function [101]. Accordingly, we sought to develop a possible rationale for how LLL may be useful as an adjunct to autologous stem cell therapy.

Table 1 depicts some of the properties of LLL that provide a rationale for the combined use with stem cells. One of the basic properties of LLL seems to be ability to inhibit inflammation at the level of innate immune activation. Representative studies showed that LLL was capable of suppressing inflammatory genes and/or pathology after administration of lipopolysaccharide (LPS) as a stimulator of monocytes [102] and bronchial cells [34], in vitro, and leukocyte infiltration in vivo [103,104]. Inflammation induced by other stimulators such as zymosan, carrageenan, and TNF-alpha was also inhibited by LLL [32,105,106]. Growth factor stimulating activity of LLL was demonstrated in both in vitro and in vivo experiments in which augmentation of FGF-2, PDGF and IGF-1 was observed [36,37,107]. Endogenous production of these growth factors may be useful in regeneration based on activation of endogenous pulmonary stem cells [108,109]. Another aspect of LLL activities of relevance is ability to stimulate angiogenesis. In COPD, the constriction of blood vessels as a result of poor oxygen uptake is results in a feedback loop culminating in pulmonary hypertension. Administration of angiogenic factors has been demonstrated to be beneficial in several animal models of pulmonary pathology [110,111]. The ability of LLL to directly induce proliferation of HUVEC cells [112], as well as to augment production of angiogenic factors such as VEGF [113], supports the possibility of creation of an environment hospitable to neoangiogenesis which is optimal for stem cell growth. In fact, a study demonstrated in vivo induction of neocapillary formation subsequent to LLL administration in a hindlimb ischemia model [114]. The critical importance of angiogenesis in stem cell mediated regeneration has previously been demonstrated in the stroke model, where the major therapeutic activity of exogenous stem cells has been attributed to angiogenic as opposed to transdifferentiation effects [115].

**Table 1:** Examples of LLL Properties Relevant to COPD

COPD Property	LLL Experiment	LLL Details	Ref
**Inflammation**	**In vivo**. Decreased joint inflammation in zymosan-induced arthritis	Semiconductor laser (685 nm and 830 nm) at (2.5 J/cm^2^)	[105]

	**In vitro**. Suppression of LPS-induced bronchial inflammation and TNF-alpha.	655 nm at of 2.6 J/cm^2^	[34]

	**In vivo**. Carrageenan-induced pleurisy had decreased leukocyte infiltration and cytokine (TNF-alpha, IL-6, and MCP)	660 nm at 2.1 J/cm^2^	[106]

	**In vitro**. LPS stimulated Raw 264.7 monocytes had reduced gene expression of MCP-1, IL-1 and IL-6	780 nm diode laser at 2.2 J/cm^2^)	[102]

	**In vivo**. Suppression of LPS-stimulated neutrophil influx, myeloperoxidase activity and IL-1beta in bronchoalveolar lavage fluid.	660 nm diode laser at 7.5 J/cm^2^	[35]

	**In vitro**. Inhibition of TNF-alpha induced IL-1, IL-8 and TNF-alpha mRNA in human synoviocytes	810 nm (5 J/cm^2^) suppressed IL-1 and TNF, (25 J/cm^2^) also suppressed IL-8	[32]

	**In vivo**. Reduction of TNF-alpha in diaphragm muscle after intravenous LPS injection.	4 sessions in 24 h with diode Ga-AsI-Al laser of 650 nm and a total dose of 5.2 J/cm^2^	[104]

	**In vivo**. Inhibition of LPS induced peritonitis and neutrophil influx	3 J/cm^2 ^and 7.5 J/cm^2^	[103]

**Growth Factor Production**			

	**In vivo**. Upregulation of TGF-β and PDGF in rat gingiva after incision.	He-Ne laser (632.8 nm) at a dose of 7.5 J/cm^2^	[36]

	**In vitro**. Osteoblast-like cells were isolated from fetal rat calvariae had increased IGF-1	Ga-Al-As laser (830 nm) at (3.75 J/cm^2^).	[107]

	**In vitro**. Upregulated production of IGF-1 and FGF-2 in human gingival fibroblasts.	685 nm, for 140 s, 2 J/cm^2^	[37]

**Angiogenesis**			

	**In vivo**. Increased fiber to capillary ratio in rabbits with ligated femoral arteries.	Gallium-aluminum-arsenide (Ga-Al-As) diode laser, 904 nm and power of 10 mW	[114]

	**In vitro**. Stimulation of HUVEC proliferation by conditioned media from LLL-treated T cells	820 nm at 1.2 and 3.6 J/cm^2^.	[119]

	**In vitro**. 7-fold increased production of VEGF by cardiomyocytes, 1.6-fold increase by smooth muscle cells (SMC) and fibroblasts. Supernatant of SMC had increased HUVEC-stimulating potential.	He:Ne continuous wave laser (632 nm). 0.5 J/cm^2 ^for SMC, 2.1 J/cm^2 ^for fibroblasts and 1.05 J/cm^2 ^for cardiomyocytes.	[113]

	**In vitro**. Direct stimulation of HUVEC proliferation	670 nm diode device at 2 and 8 J/cm^2^	[112]

**Direct Stem Cell Effects**			

	**In vivo**. LLL precondition significantly enhanced early cell survival rate by 2-fold, decreased the apoptotic percentage of implanted BMSCs in infarcted myocardium and increased the number of newly formed capillaries.	635 nm at 0.96 J/cm^2^	[120]

	**In vitro**. LLL stimulated MSC proliferation, VEGF and NGF production, and myogenic differentiation after 5-aza induction.	635 nm diode laser at 0.5 J/cm^2 ^for MSC proliferation, 5 J/cm^2 ^for VEGF and NGF production and for augmentation of induced myogenic differentiation	[121]

	**In vitro**. Increased proliferation of rat MSC.	Red light LED 630 nm at 2 and 4 J/cm(2)	[116]
	**In vitro**. Augmented proliferation of bone marrow and cardiac specific stem cells.	GA-Al-As 810 nm at 1 and 3 J/cm^2^	[117]

	**In vitro/In vivo**. Administration of LLL-treated MSC resulted 53% reduction in infarct size, 5- and 6.3-fold significant increase in cell density that positively immunoreacted to BrdU and c-kit, respectively, and 1.4- and 2-fold higher level of angiogenesis and vascular endothelial growth factor, respectively, when compared to non-laser-treated implanted cells	Ga-Al-As laser (810 nm wavelength), 1 J/cm^2^	[118]

	**In vitro**. Enhanced proliferation of adipose derived MSC in presence of EGF.	636 nm diode, 5 J/cm^2^	[55]

	**In vitro**. Enhanced proliferation and beta-1 integrin expression of adipose derived MSC.	635 nm diode laser, at 5 J/cm^2^	[54]

	**Clinical**. 660 stroke patients: 331 received LLL and 327 received sham. No prespecified test achieved significance, but a post hoc analysis of patients with a baseline National Institutes of Health Stroke Scale score of <16 showed a favorable outcome at 90 days on the primary end point (P < 0.044).	808 nm. No density disclosed.	[100]

Direct evidence of LLL stimulating stem cells has been obtained using mesenchymal stem cells derived both from the bone marrow and from the adipose tissue [116,117]. Interestingly in vivo administration of LLL stimulated MSC has resulted in 50% decrease in cardiac infarct size [118]. Clinical translation of LLL has been performed in the area of stroke, in which a 660 patient trial demonstrated statistically significant effects in post trial subset analysis [100].

## Conclusions

Despite clinical use of LLL for decades, the field is still in its infancy. As is obvious from the wide variety of LLL sources, frequencies, and intensities used, no standard protocols exist. The ability of LLL to induce growth factor production, inhibition of inflammation, stimulation of angiogenesis, and direct effects on stem cells suggests the urgent need for combining this modality with regenerative medicine, giving birth to the new field of "regenerative photoceuticals". Development of a regenerative treatment for COPD as well as for other degenerative diseases would be of considerable benefit. Regarding COPD, such treatment would be life-saving/life extending for thousands of affected individuals. Ceasing smoking or not starting to smoke would considerably impact this disease.

## Competing interests

David R Koos is a shareholder, as well as Chairman and CEO of Entest Bio. Feng Lin is research director of Entest Bio. All other authors declare no competing interest.

## Authors' contributions

FL, SFJ, DTA, FR, VB, VG, CAD, RDNC, ANP, EC, DRK contributed to literature review, analysis and discussion, synthesis of concepts, writing of the manuscript and proof-reading of the final draft.
